# Linked Consumer Registers as data infrastructure for timely and inclusive monitoring of community characteristics

**DOI:** 10.23889/ijpds.v8i2.2353

**Published:** 2023-09-14

**Authors:** Paul Longley, Justin Van Dijk, Bin Chi

**Affiliations:** 1 UCL, London, United Kingdom

We review creation and maintenance of nationwide individual level Linked Consumer Registers as DigitalFootprints Data and their use to create timely, inclusive annual neighbourhood scale research ready datasets of social and spatial mobility. Outputs include annual estimates of neighbourhood churn, neighbourhood deprivation following moves, energy usage and ‘housing career’ measures.

Individual level names and addresses are harvested from public Electoral Registers and consumer sources from 1997-2023. A novel ‘migration model’ is developed to georeference records and link them across years. The provenance of data and methods are documented in metadata to accompany derivative research ready data extracts pertaining to residential mobility occurrences and outcomes. Novel methods are developed to reveal the probable gender, ethnicity and age characteristics of all households. Data are then linked to property level Zoopla rental listings, Land Registry/Registers of Scotland transactions and energy performance statistics to link household characteristics to properties occupied before and after moves.

Results provide annual nationwide updates of neighbourhood household structure, ethnicity and demography that, subject to disclosure controls, can be honed to any convenient geography. They are validated against decennial census statistics and compared with midyear population estimates. Linkage to external datasets enables further external validation of methods used to infer moves and plug known omissions in the registers. Application of individual level demographic models makes it possible to model household structure and individual ethnic, age and gender characteristics. Summary linked and annually updated research ready datasets pertaining to neighbourhood residential churn, ethnicity, distances of residential moves housing careers and domestic energy usage are then produced.

The research is an ambitious linkage of individual and property level consumer and administrative datasets. Individual level linkage and modelling provides analytical flexibility in research ready data creation, and data linkage can be expedited for any period for which name and address data are available.

